# Volvulus du grêle sur paquet d’ascaris: à propos d’un cas

**DOI:** 10.11604/pamj.2016.24.208.8963

**Published:** 2016-07-08

**Authors:** Cheikh Diouf, Ahmed Kane, Ndeye Aby Ndoye, Oumar Ndour, Aimé Lakh Faye-Fall, Mbaye Fall, Désiré Munyali Alumeti, Gabriel Ngom

**Affiliations:** 1 Service de Chirurgie, Hôpital Régional de Ziguinchor, Université Assane Seck de Ziguinchor, Sénégal; 2 Service de Chirurgie Viscérale, Centre Hospitalier National de Nouakchott, Université des Sciences de Technologie et de Médecine de Nouakchott, Mauritanie; 3 Service de Chirurgie Pédiatrique Hôpital d’Enfants Albert Royer, Dakar, Sénégal; 4 Service de Chirurgie Pédiatrique CHU Aristide Le Dantec, Dakar, Sénégal; 5 Service de Chirurgie Centre Hospitalier Abass Ndao Dakar, Sénégal

**Keywords:** Ascaris, enfant, occlusion intestinale, résection, volvulus, Ascaris lumbricoides, child, intestinal obstruction, resection, volvulus

## Abstract

Nous rapportons un cas exceptionnel de volvulus nécrosé de l'intestin grêle dû à des ascaris adultes chez un enfant de 7 ans. A l'admission, l'enfant présentait le tableau d'occlusion intestinale qui évoluait depuis deux jours avec altération de l'état général. La radiographie de l'abdomen sans préparation retrouvait des niveaux hydroaériques de type grêlique et un aspect tigré évoquant le diagnostic d'une occlusion intestinale haute sur masse abdominale. Après la réanimation, le traitement chirurgical consistait en une laparotomie qui avait retrouvé un volvulus nécrosé de l'iléon terminale contenant des ascaris adultes. Une résection du grêle sur environ un mètre emportant le segment nécrosé suivie d'une iléostomie était réalisée. L'évolution a été favorable, l'anastomose iléo-colique fut réalisée quatre semaines plus tard. Au recul de deux ans l'enfant est indemne de tout symptôme.

## Abstract (en)

We report an exceptional case of a 7 year-old patient with necrotic small bowel volvulus due to adult ascaris lumbricoides. At the admission, the child had intestinal obstruction evolving since two days with alteration of general state. Abdominal radiography without preparation showed small bowel air-fluid levels and tiger-stripe appearance evoking the diagnosis of acute intestinal obstruction associated with abdominal mass. After resuscitation, the surgical treatment consisted of laparotomy which showed necrotic volvulus of the terminal ileum containing adult ascaris lumbricoides. The patient underwent small bowel resection, approximately one meter of affected section was removed and then an ileostomy was performed. The evolution was favorable. The patient underwent ileorectal anastomosis four weeks later. After a 2 year follow-up period the child had no symptoms.

## Introduction

L’ascaridiose, due à l’infestation de l’homme par un nématode, Ascaris lumbricoides, est la parasitose intestinale la plus fréquente dans le monde [1]. L’incidence est estimée à environ 1.000.000 de cas par an [2]. Le cycle épidémiologique est un cycle direct sans hôte intermédiaire. Les formes asymptomatiques, les plus fréquentes, sont liées à une infestation modérée. Le diagnostic de ces formes est posé lors de la découverte fortuite d’œufs dans les selles, ou lors du rejet spontané de vers adultes par l’anus. Les signes sont généralement mineurs à type de nausées, anorexie, retard pondéral mais dans 2 cas pour 1000, peut survenir une occlusion intestinale. Nous rapportons le cas d’un enfant de 7 ans qui a présenté un volvulus de l’intestin grêle sur paquet d’ascaris compliqué d’une nécrose intestinale étendue.

## Patient et observation

**Figure 1:**
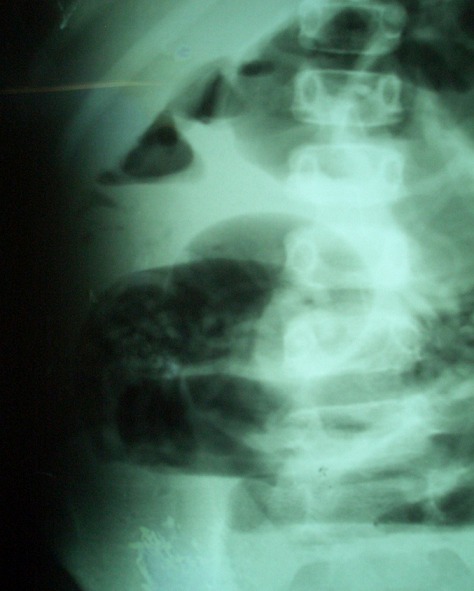
Radiographie de l’abdomen sans préparation: niveaux hydroaériques de type grêlique et un aspect tigré

**Figure 2:**
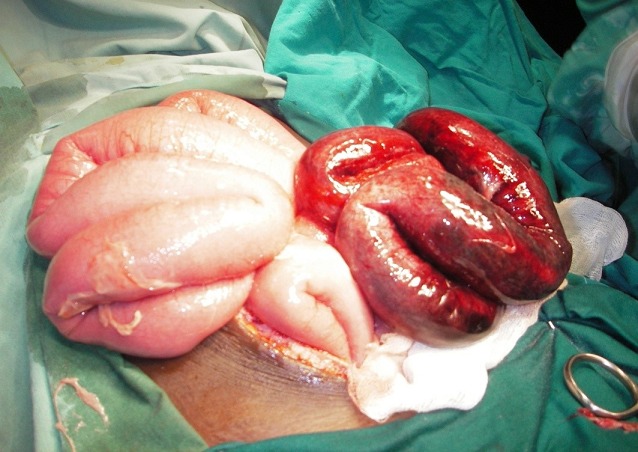
Volvulus de la dernière anse iléale avec nécrose intestinale vue per opératoire

**Figure 3:**
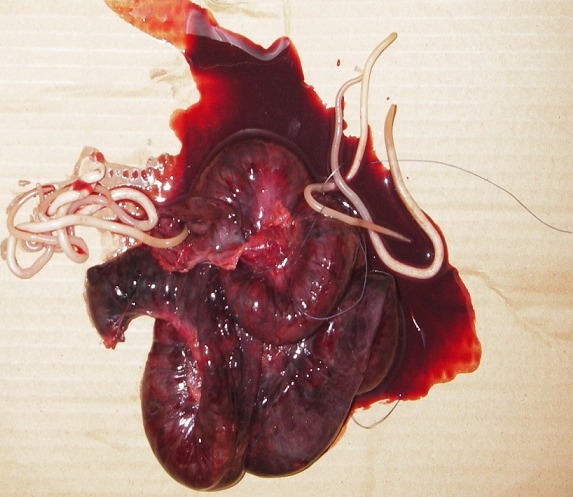
Résection de grêle emportant le segment nécrosé contenant des ascaris adultes

M. D., garçon âgé de 7 ans, sans antécédents pathologiques particuliers a été reçu dans le service des urgences chirurgicales pour une douleur abdominale ayant débutée à la fosse iliaque droite pour se généraliser à tout l’abdomen, associée à des vomissements bilieux et à un arrêt des matières et des gaz, le tout évoluant depuis 48 heures. La symptomatologie avait motivée une consultation dans un dispensaire où un traitement à base de métronidazole, d’amoxicilline et de paracétamol a été instituée. A l’examen clinique, l’enfant était déshydraté avec un mauvais état général, un faciès terreux, une température à 38°5, un pouls à 100 battements par minute. On notait aussi un discret météorisme abdominal et une douleur avec défense abdominale généralisée. Les orifices herniaires étaient libres. Le bilan paraclinique montrait une hyperleucocytose à 26910/mm^3^, des niveaux hydro-aériques de type grêlique et un aspect tigré à la radiographie de l’abdomen sans préparation (Figure 1). Après des mesures de réanimation (réhydratation, antibiothérapie, sonde nasogastrique et antalgiques), l’exploration chirurgicale montrait un volvulus de la dernière anse iléale avec nécrose des 90 derniers centimètres de grêle (Figure 2) et présence dans le grêle terminal d’une masse molle constituée de multiples structures allongées, mobiles, mesurant 15 cm en moyenne et évoquant des ascaris adultes. Il a bénéficié d’une résection d’un mètre de grêle emportant le segment nécrosé qui contenait des ascaris adultes (Figure 3) suivie d’une iléostomie. Il a reçu 400mg d’Albendazole renouvelés une semaine plus tard. Les suites opératoires ont été simples. L’anastomose iléo-iléale a été réalisée 4 semaines plus tard et l’enfant a regagné le domicile familial 5 jours après le rétablissement de la continuité digestive.

## Discussion

L'ascaridiase est la parasitose intestinale la plus fréquente dans le monde. On estime qu'un quart de la population mondiale est infectée [3] avec une prédominance dans les régions tropicales. Touchant préférentiellement l'enfant entre 2 et 10 ans [2], l'infection débute par l'ingestion des œufs embryonnés d'*Ascaris lumbricoides*. Les œufs vont donner des larves qui vont traverser la paroi jéjunale et, par voie hématogène, se retrouver au niveau des alvéoles pulmonaires où se fait leur maturation. A ce niveau, ils peuvent se manifester 5 à 25 jours après l'ingestion par un syndrome de Loeffler [1, 3] qui associe une toux, un syndrome bronchique, un infiltrat bilatéral du poumon à la radiologie et une franche hyperéosinophilie. Les ascaris migrent au niveau du carrefour aéro-digestif d'où ils sont déglutis. De retour au niveau du jéjunum, les ascaris adultes, mesurant 10 à 35 cm de long pour 2 à 6 mm de diamètre sont plus souvent latents durant leur 6 à 12 mois de vie [3]. Les symptômes sont représentés par des nausées, des vomissements, une anorexie, des douleurs abdominales sous forme de colique et, dans deux cas pour mille, une occlusion intestinale [4–6], plus fréquente entre 3 et 7 ans. Encore plus rarement, peuvent se voir une pancréatite, une cholécystite ou un abcès hépatique dus à une migration aberrante des ascaris [1, 3].

L'occlusion sur ascaridiase intestinale est généralement la conséquence d'une importante charge parasitaire [3, 5, 7]. Certains auteurs l'attribuent à une prise d'antihelminthiques qui provoque la paralysie des vers adultes et leur agglutination au niveau du grêle terminal il s'en suit une obstruction de la lumière intestinale, une invagination intestinale ou plus rarement un volvulus du grêle [3, 5, 7]. Lugaria au Kenya a trouvé un cas de volvulus du grêle sur 69 laparotomies pour ascaridiose intestinale compliquée [7]. Le diagnostic d'occlusion sur paquet d'ascaris peut être évoqué devant des antécédents de rejet d'ascaris adultes au cours de vomissements ou dans les selles. La radiographie de l'abdomen sans préparation montre, au sein des clartés digestives, des opacités linéaires [6] donnant un aspect tigré. L'échographie abdominale peut montrer le paquet d'ascaris dans le grêle sous la forme d'une masse d'échostructure mixte et constituée de multiples bandes hypoéchogènes, entrelacées, d'environ 4 mm de large et à parois dédoublées dotées de mouvements ondulatoires [8]. Bien que le scanner abdominal ne soit pas l'examen de choix pour le diagnostic de l'ascaridiase, il peut parfois visualiser les ascaris adultes dans l'intestin [3]. Le traitement de l'obstruction intestinale par paquet d'ascaris est généralement chirurgical mais, en l'absence de signes de péritonite, un traitement médical associant perfusion intraveineuse, antibiothérapie, administration de sels de Pipérazine par une sonde nasogastrique, lavement à la glycérine et à l'huile de paraffine peut donner de bons résultats et ce, même en cas d'obstruction complète [6, 9]. Le volvulus du grêle sur paquet d'ascaris impose une laparotomie. Une détorsion suivie d'une vidange antérograde du grêle, voire d'une entérotomie pour extraction des ascaris est indiquée en l'absence de nécrose intestinale [3, 8]. En cas de nécrose intestinale, une résection suivie d'une anastomose termino-terminale ou d'une stomie est réalisée. Le traitement étiologique repose sur des antihelminthiques notamment l'Albendazole. Chez notre patient, en l'absence de notion de prise d'antihelminthique avant l'apparition de la symptomatologie le volvulus serait la conséquence d'une infestation massive. La nécrose était liée à un retard diagnostic, l'enfant ayant consulté initialement dans un dispensaire. L'indication de l'exploration chirurgicale ne se discutait pas puisque l'enfant était venu dans un tableau de péritonite et que c'est l'étiologie appendiculaire qui était évoquée. L'étendue du grêle nécrosé (90 cm) est importante comparée au cas publié par Rodriguez [3]. L'iléostomie a été décidée devant l'altération de l'état général.

## Conclusion

L’'occlusion par obstruction sur paquet d’'ascaris n’'est pas rare mais l’'obstruction par strangulation est exceptionnelle. Cette complication peut être prévenue par des campagnes de déparasitage systématique en région tropicale.
